# Beyond the Skin: Topical Amphotericin B Nanocarriers Targeting Cutaneous Leishmaniasis with Suppression of Lymphatic Parasite Burden

**DOI:** 10.3390/idr18010006

**Published:** 2026-01-06

**Authors:** Francisco Alexandrino-Júnior, Gabriel Barcellos, Luiz Filipe Gonçalves-Oliveira, Luzia Monteiro de Castro Côrtes, Franklin Souza-Silva, Carlos Roberto Alves, Geovane Dias-Lopes, Juliana Figueiredo Peixoto, Beatriz Ferreira de Carvalho Patricio, Helvécio Vinícius Antunes Rocha

**Affiliations:** 1 Laboratório de Micro e Nanotecnologia, Centro de Desenvolvimento Tecnológico em Saúde (CDTS), Fundação Oswaldo Cruz, Avenida Brasil, 4036, Maré, Rio de Janeiro 21040-361, RJ, Brazil; francisco.alexandrino@fiocruz.br (F.A.-J.); gabriel.barcellos@fiocruz.br (G.B.); helvecio.rocha@fiocruz.br (H.V.A.R.); 2 Programa de Pós-Graduação em Pesquisa Translacional em Fármacos e Medicamentos, Farmanguinhos, Fundação Oswaldo Cruz, 4365, Rio de Janeiro 21041-250, RJ, Brazil; 3 Laboratório de Biologia Molecular e Doenças Endêmicas, Instituto Oswaldo Cruz, Fundação Oswaldo Cruz, Avenida Brasil, 4365, Manguinhos, Rio de Janeiro 21040-900, RJ, Brazil; luizfilipeol07@gmail.com (L.F.G.-O.); luzia@ioc.fiocruz.br (L.M.d.C.C.); calves@ioc.fiocruz.br (C.R.A.); geovane.dl@gmail.com (G.D.-L.); 4 Laboratório de Modelagem de Sistemas Biológicos, Centro de Desenvolvimento Tecnológico em Saúde, Fundação Oswaldo Cruz, Avenida Brazil, 4036, Manguinhos, Rio de Janeiro 21040-361, RJ, Brazil; franklin.frankss@gmail.com; 5 Laboratório de Pesquisa Pré-Clínica, Universidade Iguaçu, Avenida Abílio Augusto Távora 2134, Dom Rodrigo, Nova Iguaçu 26260-045, RJ, Brazil; 6 Instituto de Biologia Roberto Alcântara Gomes, Departamento de Ciências Biomédicas e Saúde, Universidade do Estado do Rio de Janeiro, Rua Arízio Gomes da Costa 186, Jardim Flamboyant, Cabo Frio 28905-320, RJ, Brazil; 7 Biomedicina, Centro de Ciências da Saúde, Universidade Católica de Petrópolis, Rua Barão do Amazonas-124, Centro, Petrópolis 25685-100, RJ, Brazil; jufpeixoto@gmail.com; 8 Laboratório de Inovação Farmacêutica e Tecnológica, Instituto Biomédico, Universidade Federal do Estado do Rio de Janeiro (UNIRIO), Rio de Janeiro 20211-005, RJ, Brazil

**Keywords:** nanoparticle, skin, lymph node

## Abstract

Background/Objectives: Cutaneous leishmaniasis (CL) remains a global health challenge, with treatment options often limited by drug resistance and systemic toxicity. Amphotericin B (AmB) represents a promising alternative. but intravenous administration causes severe systemic adverse effects. Despite growing interest in topical therapies, knowledge gaps remain regarding the comparative efficacy of delivery systems, including the influence of treatment timing and potential intrinsic effects. This study aimed to develop and characterize different topical AmB formulations (polymeric nanoparticles (PCL-AmB), a lipid-based (Oil_AmB) formulation, and a gel emulsion) to evaluate their in vivo efficacy against CL in a murine model, considering treatment initiation timing and potential intrinsic effects of the delivery systems. Methods: Formulations were prepared and characterized in terms of hydrodynamic size, polydispersity index, and AmB content. Antileishmanial activity was assessed in two independent in vivo experiments, with topical monotherapy administered five days per week for four weeks, starting either 10 or 30 days post-infection, representing early and established chronic stages of infection, respectively. Results: All formulations exhibited nanoscale dimensions and high homogeneity, with the lipid system demonstrating superior AmB solubilization. Both PCL-AmB and Oil_AmB reduced parasite load in the footpad, with Oil_AmB also reducing parasite load in draining lymph nodes. Conclusions: PCL-AmB and Oil_AmB reduced lesions and parasite burden in *L. amazonensis*-infected mice. Treatment timing was critical, with early Oil_AmB also reducing parasite loads in draining lymph nodes. These findings suggest that topical AmB formulations may provide a promising alternative for CL treatment, though further studies are required to optimize efficacy and administration schedules.

## 1. Introduction

Leishmaniases are vector-borne parasitic diseases caused by protozoa of the genus *Leishmania* and transmitted to humans by infected sandflies of the Psychodidae family [1]. More than twenty *Leishmania* species and subspecies have been identified to date, and over ninety sandfly species are known vectors [2]. Depending on the parasite–host interaction, transmission may be anthroponotic or zoonotic, ultimately resulting in three primary clinical forms of leishmaniasis: visceral, cutaneous, and mucocutaneous [2,3].

Leishmaniasis is classified by the World Health Organization (WHO) as a neglected tropical disease, with an estimated 700,000 to 1 million new cases annually and up to 1.6 million disability-adjusted life years lost worldwide [2,4]. Cutaneous leishmaniasis (CL) is the most common clinical presentation. It typically begins with a papule at the site of inoculation, usually in exposed body areas, which may progress to a plaque or nodule and eventually form an ulcer [5]. Lesion number varies widely, and the parasite may spread through the lymphatic system, leading to lymphadenopathy, satellite lesions, or sporotrichoid lymphangitis [6,7]. Atypical presentations, including eczematous, erysipeloid, lupoid, or verrucous lesions, may also occur [8,9].

Among the eleven countries responsible for most global CL notifications, Brazil remains the leading contributor in the Americas [10]. CL occurs in all Brazilian states as an endemic condition, reflecting a persistently high transmission risk predominantly associated with *Leishmania* (*Leishmania*) *amazonensis*, *L.* (*Viannia*) *guyanensis*, and *L.* (*V.*) *braziliensis* [11]. The national epidemiological scenario is further aggravated by delayed diagnosis, high morbidity, socioeconomic vulnerability, HIV coinfection, therapeutic limitations, and underreporting [12,13,14,15]. Together, these factors underscore the need for new strategies to improve disease control and expand the available therapeutic arsenal.

The treatment of CL remains challenging because no universally effective therapeutic approach or preventive measure, such as a vaccine, is currently available [16,17]. Treatment decisions must consider multiple factors, including parasite species, clinical presentation, and host immune status [18]. Several therapeutic strategies have been investigated, ranging from monotherapies to combination regimens. However, some interventions—such as azithromycin or thermotherapy—have shown limited benefit or even reduced healing rates [19], while others, including imiquimod or pentoxifylline, provide minimal advantage when combined with antimonials [19].

Antimonials at 20 mg/kg for 28–30 days have remained the standard of care for decades, yet increasing resistance has reduced their effectiveness [20,21]. These drugs may also cause adverse effects, ranging from injection-site pain and gastrointestinal discomfort to cardiac arrhythmias, QTc prolongation, and ventricular tachycardia [22]. Patients coinfected with HIV experience more severe toxicity, reduced response rates, and increased mortality [23,24]. To mitigate systemic toxicity, topical approaches such as intralesional (IL) antimonials and paromomycin ointment have been explored. However, their therapeutic outcomes vary substantially: IL therapy shows cure rates ranging from no measurable improvement over placebo [25] to approximately 70% (95% CI: 52–83%) [26], and paromomycin ointment demonstrates highly inconsistent efficacy across clinical trials [27]. The true effectiveness of these treatments remains difficult to determine, as systematic reviews by Heras-Mosteiro et al. and Brito et al. have identified both high variability in outcomes and low methodological quality in the available studies [27,28].

In light of this scenario, the development of safe and effective treatment for CL is required. From this perspective, there is growing evidence of the effectiveness of Amphotericin B (AmB) as an alternative therapy [29,30], which exhibits few reports about therapeutic failure or relapse [30]. Conversely, this molecule exhibits troublesome physical-chemical characteristics, e.g., low aqueous solubility in physiological pH [31], autooxidation [32], high molecular weight, and poor oral permeability (violating three out of five Lipinski’s rules [31,33] and being characterized as class IV in the Biopharmaceutical Classification System [34,35]). These properties collectively contribute to its limited oral bioavailability, restricting its clinical use via intravenous administration.

This approach has been associated with notably severe toxic adverse effects, with nephrotoxicity being among the most severe [36]. This harmful effect is, in part, attributed to the direct toxic interaction of AmB with renal tubular cells [37]. A promising strategy to overcome these challenges and enable outpatient treatment involves the development of a topical formulation with properties that surpass these challenging physical-chemical properties of AmB. Numerous drug delivery systems have diligently been developed to pursue this goal, with lipid and polymeric systems emerging as prominent candidates.

Nevertheless, despite the growing interest in topical therapies for CL, a significant knowledge gap remains regarding the comparative efficacy of polymeric or lipid-based delivery systems for AmB in enhancing cutaneous penetration, improving local bioavailability, and achieving effective parasite clearance through topical administration. This gap is further accentuated by the limited understanding of how treatment timing influences therapeutic outcomes and whether the delivery systems themselves contribute to immunomodulatory effects. This scenario restricted the development of safer alternatives to systemic AmB.

Therefore, it was hypothesized that polymeric and lipid-based topical formulations could overcome these physicochemical limitations of AmB, enhance local drug availability, and improve therapeutic outcomes in cutaneous leishmaniasis. For this reason, the objective of the present study was to develop and characterize different polymeric and lipid-based formulations loaded with AmB and assess their therapeutic performance in a validated murine model of CL, considering the impact of treatment initiation timing and potential intrinsic effects of the delivery systems.

## 2. Materials and Methods

### 2.1. Materials

Amphotericin B (AmB; purity 89.6%) was obtained from Hangzhou Dayangchem (Hangzhou, China). Phosal^®^ 50 PG and Imwitor^®^ 308 were purchased from Lipoid (Ludwigshafen, Germany) and IOI Oleo GmbH (Hamburg, Germany), respectively. Wizard Genomic DNA Purification Kit was purchased from Promega (Madison, WI, USA). Schneider’s insect medium pH 7.2 and fetal bovine serum were obtained from Thermo Fisher Scientific (Waltham, MA, USA). Poloxamer 407 (Kolliphor^®^ P407) was kindly donated by BASF (Ludwigshafen, Germany). Polysorbate 80 (Super Refined Polysorbate 80) was provided by Croda (Campinas, Brazil). Acetone and hydrochloric acid were obtained from Biograde (Anápolis, Brazil). Polycaprolactone (PCL, Mw 14,000), penicillin, streptomycin, Span^®^ 80, dichloromethane (DCM), acetonitrile (ACN), ethanol, and all other analytical-grade chemicals and solvents were purchased from Sigma-Aldrich (St. Louis, MI, USA) or Merck (Darmstadt, Germany).

### 2.2. Preparation of AmB-Loaded and Placebo Polymeric Nanoparticles

AmB-loaded PCL nanoparticles (PCL–AmB) were prepared by nanoprecipitation as previously described by Souza et al. [38], with minor modifications. First, a polymer solution was obtained by dissolving 16 mg of PCL in 3 mL of acetone acidified with 30 μL of 0.1 N HCl at 40 °C under magnetic stirring (RT 15 Power, IKA, Staufen im Breisgau, Germany). In parallel, 4 mg of AmB were dissolved in 400 μL of DMSO, followed by the addition of 1 mL of MeOH to obtain a homogeneous drug solution.

The organic phase was added dropwise to 12 mL of an aqueous phase containing 0.3% *w*/*v* polysorbate 80 under continuous magnetic stirring (C-MAG HS-7, IKA, Staufen im Breisgau, Germany) at room temperature. After 10 min of mixing, organic solvents were removed under reduced pressure at 37 °C for 30 min using a rotary evaporator (RV9, IKA, Staufen im Breisgau, Germany).

Unencapsulated AmB was removed by centrifugation (Megafuge 16, Thermo Fisher Scientific, Waltham, MA, USA) at 985× *g* for 1 h at room temperature. Placebo nanoparticles were produced following the same procedure without AmB.

For in vivo use, nanoparticles were frozen at −20 °C for 16 h in the presence of poloxamer 407 (cryoprotectant) and then lyophilized at −35 °C for 48 h (Alpha 2-4 LD plus, Christ, Osterode am Harz, Germany.). Lyophilized nanoparticles were reconstituted in ultrapure water at a concentration of 16% *w*/*v*. All formulations were prepared in triplicate.

### 2.3. Preparation of AmB-Loaded Gel Emulsions

Self-nanoemulsifying AmB-loaded gel emulsions were prepared as previously reported [39], with modifications. A lipid mixture was prepared by combining Phosal^®^ 50 PG (75% *w*/*w*), Imwitor^®^ 308 (18% *w*/*w*), Span^®^ 80 (1% *w*/*w*), and ethanol (6% *w*/*w*). An excess of AmB (3 g) was added, and the mixture was homogenized at 250 rpm and 25 ± 0.1 °C for 24 h in an incubator shaker (KS 4000 IC, IKA, Staufen im Breisgau, Germany).

The suspension was centrifuged at 14,500× *g* for 30 min, and the supernatant was recovered. This procedure was repeated until no AmB pellet remained. Drug content in the final supernatant was quantified as described in Section 2.4.

To obtain the gel emulsion, an aqueous solution containing 16% *w*/*w* poloxamer 407 was added to the lipid phase at a 3:1 ratio (gel:lipid) under gentle stirring until complete homogenization. Placebo formulations were prepared without AmB. All formulations were produced in triplicate.

### 2.4. Quantification of AmB in Nanoemulsions and Nanoparticles

AmB content in lipid-based formulations was quantified by UV–visible spectrophotometry using derivative spectrophotometry, as previously described [39]. Samples were diluted in DCM/DMSO (8:2, *v*/*v*), and spectra were recorded from 300 to 450 nm with a 0.1 nm step size (UV-1800, Shimadzu, Kyoto, Japan). The first derivative was calculated, and absorbance at 417 nm was quantified using a validated calibration curve in DCM/DMSO.

For PCL–AmB nanoparticles, 500 μL of formulation was placed into a 100 kDa MWCO centrifugal filter (Amicon^®^ Ultra, Merck (St. Louis, MI, USA)) and centrifuged at 7500× *g* for 20 min. The retained fraction was recovered and dissolved in ACN/DMSO (6:4, *v*/*v*). UV–visible spectra were recorded from 300 to 450 nm, and derivative absorbance at 411 nm was quantified using a validated calibration curve prepared in ACN/DMSO.

### 2.5. Characterization of Hydrodynamic Size and Polydispersity Index

Hydrodynamic diameter and polydispersity index (PDI) of gel emulsions and PCL nanoparticles were measured by dynamic light scattering (DLS) using a Zetasizer 3000 (Malvern Instruments, Worcestershire, UK), equipped with a photon correlation spectrometer and a 632 nm laser at a 90° scattering angle. Measurements were performed at 25 °C for nanoparticles and 37 °C for the gel emulsion due to its thermoresponsive behavior [40]. Results represent the mean of three measurements, each consisting of 15 scans.

### 2.6. In Vivo Experimental Design

The in vivo antileishmanial efficacy of the gel emulsion and PCL–AmB formulations was evaluated in two independent experiments, each with five animals per group. The sample size was determined based on an expected effect size (Cohen’s d) corresponding to an approximately 60% reduction in parasite load, with a significance level (α) of 0.05 and a statistical power (1-β) of 80%. Animals showing lesion volumes ≥ 3 mm^3^, necrosis, ulceration, metastatic signs, or signs of excessive distress, such as impaired mobility or severe tissue damage, were excluded according to criteria established a priori in the approved ethical protocol (CEUA: L0038/19). No animals met these criteria, and all planned data points were included in the final analysis.

Although the mice were isogenic, allocation to cages was performed without intentional selection to minimize bias. Each animal was individually marked with a non-toxic permanent marker to reduce potential confounders. Group allocation was known to the personnel involved during allocation, experimental procedures, outcome assessment, and data analysis.

In the first experiment, treatment began 30 days after infection. Based on these results, it was hypothesized that the viscosity of the poloxamer-based aqueous phase, combined with the poor aqueous solubility of AmB, could limit transdermal permeation. To test this, a second experiment was conducted using only the oil phase of the gel emulsion (Oil_AmB), collected immediately after centrifugation and before incorporation into the gel matrix, with treatment starting 10 days post-infection. The primary outcome measure for sample size calculation was the therapeutic efficacy of the formulations, assessed by reduction in lesion volume. Parasite load quantified by real-time PCR was considered a secondary outcome to support interpretation of treatment effects.

### 2.7. Parasite Culture and Maintenance

*Leishmania* (*Leishmania*) *amazonensis* (MHOM/BR/73/LTB0016) was maintained through serial passage in BALB/c mouse footpad lesions. After in vitro isolation, promastigotes were cultured at 28 °C in Schneider’s medium pH 7.2 (Thermo Fisher Scientific, Waltham, MA, USA) supplemented with 10% fetal bovine serum (Gibco, Thermo Fisher Scientific, Waltham, MA, USA), penicillin (100 IU/mL), and streptomycin (100 µg/mL) (Sigma-Aldrich, St. Louis, MI, USA). Parasites in the stationary phase were used for infection assays.

### 2.8. Experimental Mouse Infection and Treatment Schedules

Female BALB/c mice (*Mus musculus*), inbred strain, aged 6–8 weeks and weighing approximately 20 g, were obtained from the Instituto de Ciência e Tecnologia em Biomodelos (ICTB/Fiocruz, Rio de Janeiro, Brazil). All animals were healthy, immunocompetent, maintained under specific pathogen-free conditions, and had no prior experimental manipulation. Animals were housed in polycarbonate micro-isolator cages (30 × 20 × 23 cm; max. five mice per cage) on ventilated racks with HEPA-filtered airflow (Alesco, São Paulo, Brazil), with a 12 h light/dark cycle and controlled temperature (21 ± 1 °C). Bedding, feed, and water were autoclaved and provided ad libitum. Mice underwent a standard acclimatization period before the start of experiments.

Promastigotes were harvested, washed twice in phosphate-buffered saline (PBS; 10 mM, pH 7.2) at 3800× *g* for 10 min at 4 °C, and resuspended in PBS. Animals were inoculated with 50 μL of a suspension containing 1 × 10^4^ promastigotes into the left hind footpad using a sterile syringe and needle.

Lesion development was monitored by measuring footpad height and width with a digital caliper. Lesion volume (mm^3^) was calculated as the difference between the infected and contralateral (right) footpad.

Two treatment protocols were evaluated. In the first experiment, mice received either the gel emulsion or the PCL–AmB formulation, each with its corresponding placebo group. In the second experiment, animals were treated with Oil_AmB or PCL–AmB. All formulations were applied topically to the footpad five times per week for four weeks (20 applications). Following topical application, the treated mice were briefly placed in a clean cage without bedding. This prevented contact of the treated footpad with absorbent materials and ensured adequate exposure time for proper absorption of the preparation. The animals remained in this cage for a period sufficient to allow complete drying and adherence of the formulation to the skin surface. Treatment started when lesion volume reached ≥0.5 mm^3^ (first experiment, day 30) or ≥0.4 mm^3^ (second experiment, day 10), representing early and established chronic stage of infection, respectively. Each application contained ~70 μg AmB.

### 2.9. Determination of Parasite Load

Parasite burden was quantified by real-time PCR (qPCR) targeting kinetoplast DNA (kDNA). DNA was extracted from footpad tissue and draining lymph nodes using the Wizard Genomic DNA Purification Kit (Promega, Madison, WI, USA) and quantified with a NanoDrop 2000c spectrophotometer (Thermo Scientific, Waltham, MA, USA).

Primers targeted parasite kDNA1 (forward: 5′-GGGTAGGGGCGTTCTGC-3′; reverse: 5′-TACACCAACCCCCAGTTTGC-3′) and mouse β-actin (forward: 5′-AGAGGGAAATCGTGCGTGAC-3′; reverse: 5′-CAATAGTGATGACCTGGCCGT-3′) [41,42,43].

Amplification was performed on a 7500 Fast Real-Time PCR system (Applied Biosystems, Foster City, CA, USA) using GoTaq™ PCR Master Mix (Promega), 250 nM (kDNA1) or 100 nM (β-actin) primers, and 12.5 ng DNA per reaction. Cycling conditions were: 95 °C for 5 min; 40 cycles of 95 °C for 15 s and 60 °C for 1 min; followed by a melting curve. Parasite load was estimated from a standard curve generated with 10-fold serial dilutions of *L.* (*L.*) *amazonensis* DNA (100 ng to 1 pg).

### 2.10. Statistical Analysis

Data are expressed as mean ± standard deviation and were analyzed using GraphPad Prism 8.0 (GraphPad Software, San Diego, CA, USA). Hydrodynamic size was compared using an unpaired *t*-test with Welch’s correction. Encapsulation efficiency was analyzed by Kruskal–Wallis followed by Dunn’s test. Differences between in vivo groups were evaluated using one-way ANOVA followed by Tukey’s post hoc test. All datasets were evaluated for normality and homogeneity of variances. Effect sizes and 95% confidence intervals were calculated to quantify the magnitude and precision of differences between groups. Statistical significance was defined at *p* ≤ 0.05.

### 2.11. Ethical Approval

All animal procedures were approved by the Committee for the Ethical Use of Animals of Instituto Oswaldo Cruz (L0038/19, approved on 10 December 2019). Access to *L.* (*L.*) *amazonensis* was registered in the Brazilian System of Genetic Resource Management and Associated Traditional Knowledge (SisGen) under number A39FF58 (approved on 22 February 2022).

## 3. Results

### 3.1. Determination of Hydrodynamic Size and Polydispersity Index

**Figure 1:**
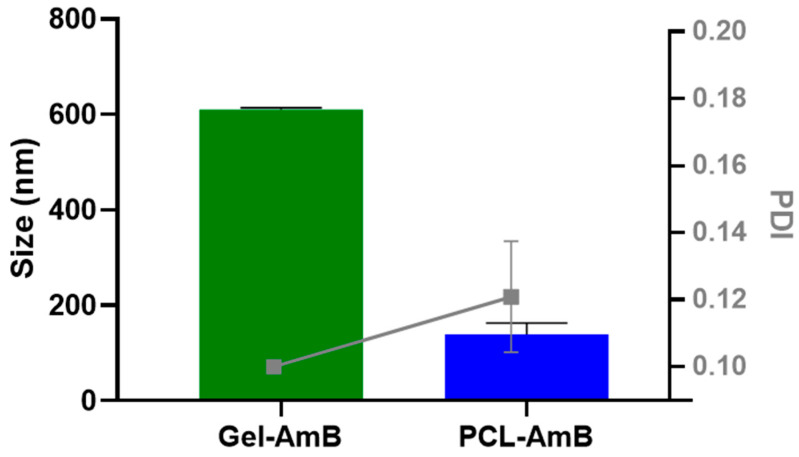
The Amphotericin B-loaded poly(ε-caprolactone) nanoparticles (PCL–AmB) exhibited an average hydrodynamic diameter of 140 ± 23 nm and a polydispersity index (PDI) of 0.12 ± 0.02. In contrast, the gel emulsion formulation was found to be significantly larger (*p* < 0.0001), with an average hydrodynamic diameter of approximately 610 ± 4 nm and a PDI of 0.10 ± 0.00. All data represent the mean ± standard deviation (*n* = 3).

The hydrodynamic size and polydispersity index (PDI) of Amphotericin B-loaded poly(ε-caprolactone) nanoparticles (PCL-AmB) and lipid-based gel emulsion formulations were determined by dynamic light scattering. As shown in Figure 1, PCL-AmB nanoparticles exhibited an average hydrodynamic diameter of 140 ± 23 nm and a PDI of 0.12 ± 0.02. In contrast, the lipid system also displayed a nanoscale size but was significantly larger (*p* < 0.0001), with an average diameter of approximately 610 ± 4 nm and PDI of 0.10 ± 0.00, more than four times greater than that of the polymeric nanoparticles.

### 3.2. Quantification of AmB in the Nanocarriers

**Figure 2:**
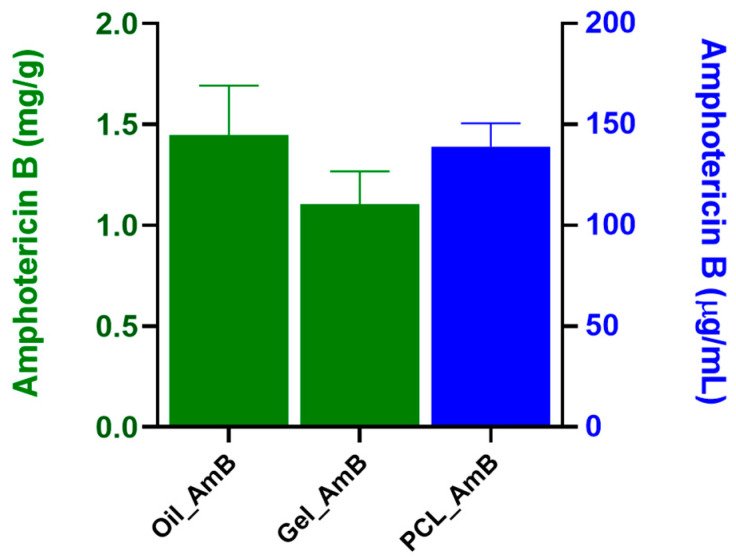
Amphotericin B content in lipid and polymeric formulations. The lipid-based systems (Oil_AmB and Gel_AmB) showed high AmB solubilization (1.45 ± 0.24 mg/g and 1.1 ± 0.16 mg/g, respectively) with no statistical difference in AmB solubility (*p* = 0.356). Conversely, the initial polymeric nanoparticles had significantly lower concentration (0.14 ± 0.12 mg/mL, *p* < 0.005), but after lyophilization and re-suspension, the concentration reached approximately 1.42 mg/mL, a level comparable to the lipid mixture. Data represent mean ± standard deviation (*n* ≥ 3), and concentrations are expressed as mg/g (green bars) and µg/mL (blue bars).

The solubilization capacity of the formulations for Amphotericin B (AmB) was assessed (Figure 2). The lipid mixture demonstrated notable solubilization, reaching 1.45 ± 0.24 mg/g. The addition of the gel to the oil phase (Oil_AmB) did not significantly alter AmB solubility (1.1 ± 0.16 mg/g, *p* = 0.356). In contrast, polymeric nanoparticles exhibited considerably lower AmB concentration (0.14 ± 0.12 mg/mL, *p* < 0.005). However, after re-suspending the polymeric lyophile, the achieved concentration was equivalent to that of the lipid mixture (approximately 1.42 mg/mL).

### 3.3. Efficacy Assessment of Amphotericin B Preparations

**Figure 3:**
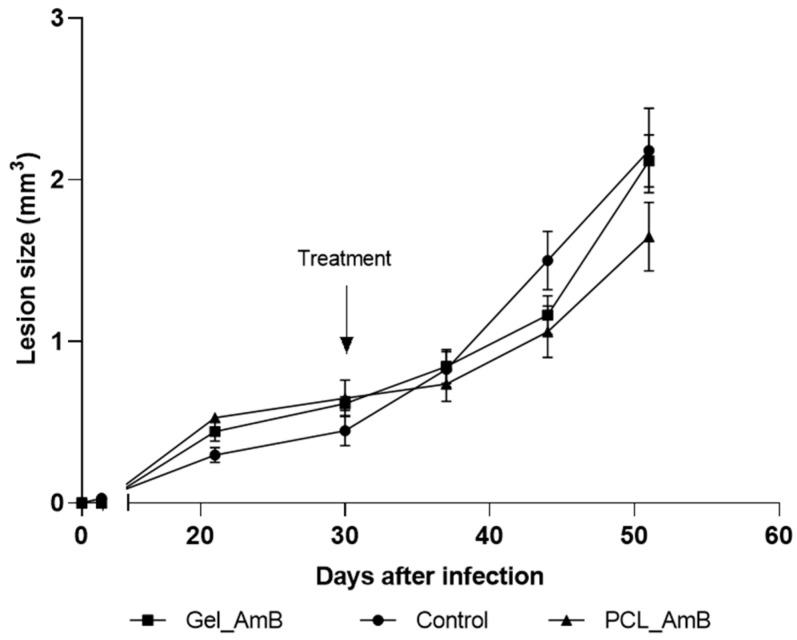
Evolution of footpad lesions in mice infected with *L.* (*L.*) *amazonensis*. Promastigotes were inoculated in the left hind footpad of BALB/c mice as described in Section 2. Materials and Methods. After 30 days of infection (indicated by the arrow), mice were treated topically for 20 days with gel emulsion and PCL-AmB (~70 µg AmB). Lesion sizes (mm^3^) were measured up to the 50th day post-infection. Results represent the means ± standard deviation of one experiment. Lesion size curves of treated groups were compared to those of the untreated control group (*n* = 5).

The initial topical treatment, applied for four weeks post-infection, resulted in significant reductions in lesion sizes only in the group treated with PCL-AmB monotherapy. Specifically, a 12.7% decrease in lesion size was observed compared to the untreated control group at week 6 post-infection. Conversely, no apparent reduction in lesion size was observed for the gel emulsion-treated group (Figure 3).

**Figure 4:**
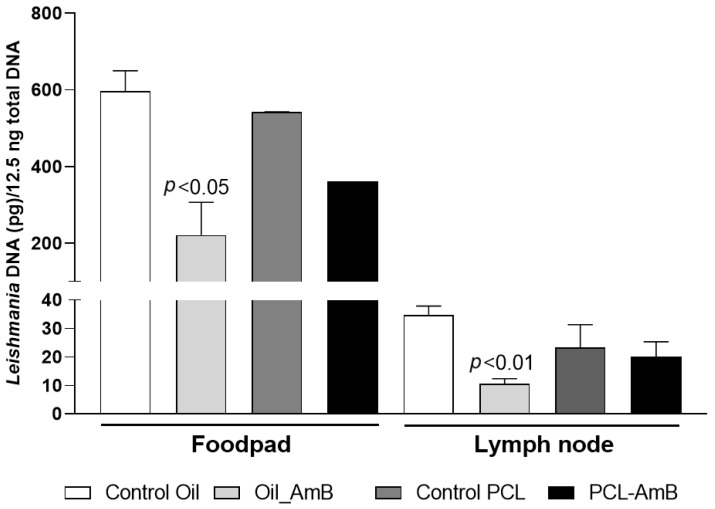
Parasite load in footpad lesions and draining lymph nodes of BALB/c mice on day 30 post-infection. Parasite load was determined by quantitative real-time PCR assays using DNA extracted from footpad and lymph node samples collected at the experimental endpoint (6 weeks post-infection) from mice infected with *L.* (*L.*) *amazonensis*. Topical treatment of the infected footpad with gel emulsion and PCL-AmB (~70 µg AmB) was administered for 20 days and compared to the untreated control group. Parasite load was expressed as *Leishmania* DNA per 12.5 ng of total DNA and is presented as the mean ± standard deviation from at least three animals per group. Statistical differences compared to the respective control were analyzed using Student’s *t*-test.

A molecular method was employed to quantify parasite load in both treated and untreated mice, using qPCR targeting *L.* (*L.*) *amazonensis* kinetoplast DNA (kDNA). Quantification was conducted on samples collected from the footpad and lesion-draining lymph nodes (Figure 4). The results demonstrated a significant reduction (*p* < 0.05) in parasite load in treated mice. This reduction was more pronounced in the footpad, where residual parasite loads were approximately 20% and 9% of the untreated control for gel emulsion and PCL-AmB, respectively. In contrast, no significant decrease was observed in draining lymph nodes after treatment with either formulation.

**Figure 5:**
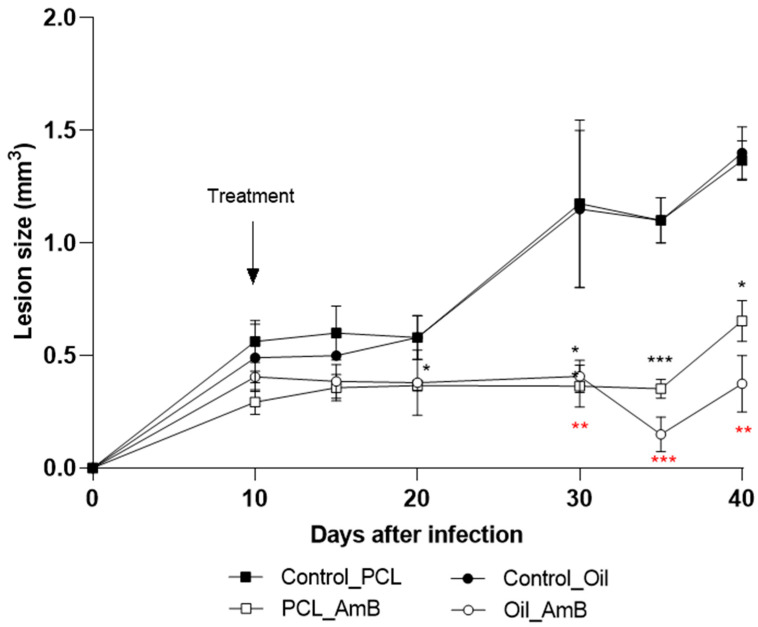
Evolution of footpad lesions in BALB/c mice infected with *L.* (*L.*) *amazonensis*. Promastigotes were inoculated into the left hind footpad of BALB/c mice, as described in Section 2. Materials and Methods. Ten days post-infection (arrow), mice were topically treated for ten days with Oil_AmB and PCL-AmB (~70 µg AmB), alongside their respective controls. Lesion size (mm^3^) was measured up to day 40 post-infection. Results are presented as mean ± standard deviation from one experiment with at least three animals per group. Lesion sizes were compared with the corresponding control groups: Control_Oil (red asterisk) and Control_PCL (black asterisk), using Student’s *t*-test. Statistical significance: *p* < 0.05 (*), *p* < 0.01 (**), and *p* < 0.001 (***). The raw data is in the Supplementary Materials.

A second treatment approach was investigated using PCL-AmB and Oil_AmB, with therapeutic intervention initiated 10 days post-infection in murine subjects. This strategy aimed to delay lesion progression from early stages of infection (Figure 5). Both AmB formulations effectively postponed lesion development compared to the control group. A significant reduction in footpad lesion size was observed on day 30 (70% for Oil_AmB: *p* = 0.0046; 60% for PCL-AmB: *p* = 0.0044), day 35 (85% for Oil_AmB: *p* = 0.0006; 70% for PCL-AmB: *p* = 0.0006), and on day 40 (77% for Oil_AmB: *p* = 0.0298; 72% for PCL-AmB: *p* = 0.0068). Notably, only Oil_AmB treatment led to a significant reduction (45%) on day 20 (*p* = 0.0335).

**Figure 6:**
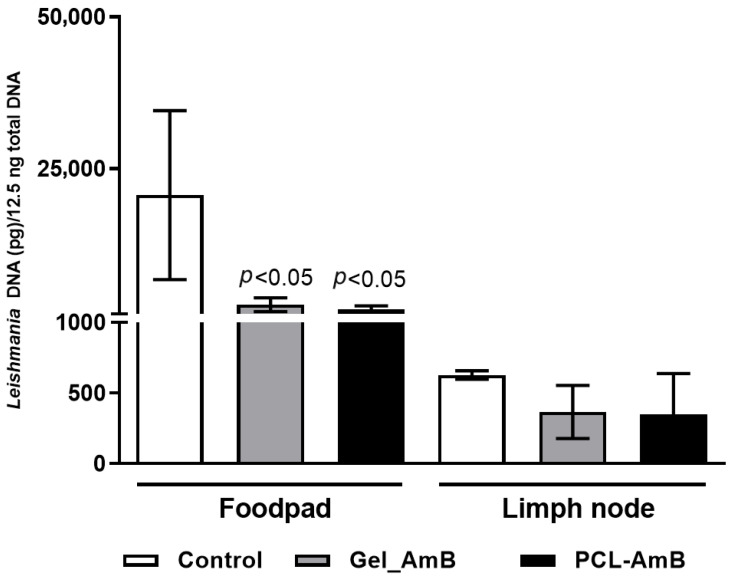
Parasite load in footpad lesions and draining lymph nodes of BALB/c mice treated 10 days after infection. DNA was extracted from footpad and lymph node samples collected on day 30 post-infection from BALB/c mice infected with *L.* (*L.*) *amazonensis* and treated with Oil_AmB and PCL-AmB (~70 µg AmB), as well as from the respective control groups. Parasite load was expressed as *Leishmania* DNA per 12.5 ng of total DNA. Results are presented as mean ± standard deviation from two independent experiments with at least three animals per group. Statistical significance compared to the control group was analyzed using Student’s *t*-test. The raw data is in the Supplementary Materials.

Parasite load in footpads and lesion-draining lymph nodes from these treatment groups was also assessed. Results showed a reduction in parasite load in footpads (63% for Oil_AmB; 34% for PCL-AmB) and in draining lymph nodes (70% for Oil_AmB; 10% for PCL-AmB) compared to the respective control groups. A statistically significant decrease was detected in both footpads and lymph nodes for mice treated with Oil_AmB (Figure 5 and Figure 6).

These findings indicate that PCL-AmB effectively reduces lesion size and parasite burden at the infection site, whereas Oil_AmB accelerates lesion control and limits parasite dissemination, highlighting the impact of formulation composition on topical efficacy.

## 4. Discussion

In this study, we developed and characterized two AmB–based topical formulations and evaluated their therapeutic potential in a murine model of CL. The combined analysis of physicochemical properties and biological responses provides important insights into how formulation design influences in vivo performance.

Both formulations presented PDI values below 0.15, indicating a high degree of homogeneity regardless of the production method. The hydrodynamic size of the PCL nanoparticles was consistent with previous findings by Marcano et al. [44], confirming the reproducibility of the preparative procedure. As expected for systems produced via bottom-up strategies, PCL nanoparticles were substantially smaller than the droplets of the gel emulsion, which relies on a top-down process. This difference reflects the distinct formation mechanisms: nanoprecipitation favors rapid nucleation of polymeric nuclei, generating smaller structures [45], whereas emulsification often results in broader droplet size distributions depending on shear forces and interfacial tension [46]. Nevertheless, there are no universal patterns in the literature [47,48] indicating that compositional factors and process parameters can invert this trend, reinforcing the need for case-by-case optimization.

Regarding drug incorporation, the dissolution method used for the lipid system was more efficient than the entrapment strategy applied to PCL nanoparticles, as highlighted in Figure 2. Interestingly, after lyophilization and resuspension, the AmB concentration in the polymeric formulation increased to levels comparable to those of the lipid mixture (1.42 mg/mL). This suggests that freeze-drying contributed not only to drug stabilization but also to a concentration effect, possibly due to reduced aqueous volume and improved drug–polymer interactions following matrix reorganization during dehydration. These observations highlight the importance of post-processing in determining the final drug dosage of nanoformulations [49].

In vivo, both formulations showed promising therapeutic activity in BALB/c mice infected with *L.* (*L.*) *amazonensis*, a model known to mimic severe and difficult-to-treat CL [50,51,52]. Reductions in lesion size and parasite burden were observed even when treatment was initiated 10 days post-inoculation, a stage characterized by well-established pathology. These outcomes, observed in a stringent, non-self-healing model, indicate that the formulations are active in reducing lesion size and parasite burden. While systemic and topical treatment options for CL remain limited, these results provide preliminary evidence that polymeric and lipid-based topical systems may offer a basis for further preclinical exploration.

Treatment timing played a critical role: earlier intervention (10 days post-infection) resulted in greater reductions in parasite loads in the draining lymph nodes, particularly for PCL-AmB (Figure 4 and Figure 6). This may reflect the higher propensity of nanoparticles to penetrate early-stage lesions or to modulate early inflammatory responses [53,54]. Notably, the drug-free formulations also reduced parasite burden compared with untreated controls (Supplementary Materials), suggesting a potential contribution of the delivery system itself to local immunomodulation [55,56].

Despite these encouraging results, complete lesion resolution was not achieved. This indicates that, although the formulations show potential, additional optimization may be required to achieve full infection control. Factors such as drug loading, kinetics release, application frequency, and combination or sequential therapies could be explored to enhance therapeutic outcomes [57]. Moreover, linking physicochemical findings to therapeutic outcomes, for example, by assessing whether particle size or encapsulation efficiency influences skin penetration or local drug bioavailability, could provide important insights into formulation performance [58,59].

This study has some limitations. Experiments were conducted exclusively in BALB/c mice infected with *L. amazonensis*, a rigorous model that cannot fully reproduce the clinical heterogeneity of human CL. Caution is warranted when extrapolating these findings to CL caused by other *Leishmania* species. Additional limitations include the evaluation of only selected concentrations and AmB formulations, thereby requiring further dosage optimization. Moreover, the lack of local pharmacokinetic and tissue distribution data limits mechanistic interpretation of the observed outcomes.

Overall, the results indicate that both nanoformulations may hold potential as topical candidates for CL. Future research should focus on optimizing formulation parameters, evaluating combination strategies, and studying biodistribution and local pharmacokinetics to better understand their therapeutic potential.

The experimental infection protocol adhered to the Basic Ethical Principles for Research and Experimentation with Animals. Lesion volumes were carefully monitored to ensure they did not exceed 3 mm^3^, and measures were taken to prevent necrosis, ulcers, or undue suffering.

## 5. Conclusions

In conclusion, both Oil_AmB and PCL-AmB formulations showed promising activity in reducing footpad lesions and parasite load in the BALB/c-*L.* (*L.*) *amazonensis* model. These results provide preliminary evidence that polymeric and lipid-based topical systems are promising candidates for enhancing local AmB delivery and exerting antileishmanial effects.

A particularly noteworthy aspect of this investigation was the critical influence of treatment timing and formulation composition on overall efficacy and specific outcome measures. For established chronic infections (30 days post-infection), PCL-AmB and the gel emulsion reduced footpad parasite load but did not significantly impact draining lymph node burden. Conversely, earlier intervention (10 days post-infection) yielded more comprehensive benefits. During early treatment, both PCL-AmB and Oil_AmB delayed lesion progression and reduced footpad sizes. However, only early Oil_AmB significantly reduced parasite loads in both footpads and draining lymph nodes, suggesting a potential limitation in controlling parasite dissemination in well-established infections. These findings imply that future CL treatment protocols could significantly benefit from early diagnostic and therapeutic interventions, particularly with formulations designed for effective deep tissue penetration.

Furthermore, the observed reduction in parasite burden by drug-free formulations in comparison to untreated controls might suggest a potential intrinsic immunomodulatory effect exerted by the delivery systems themselves, possibly by influencing local inflammatory responses or modulating the host’s immune cells. This novel insight warrants further investigation into the specific mechanisms involved, as it could open new avenues for developing synergistic or even independent therapeutic strategies centered on nanocarrier-mediated immunomodulation.

Nevertheless, it is worth mentioning that these conclusions are specific to the *L. amazonensis*-infected BALB/c mouse model. While robust, this model does not fully represent the heterogeneity of human CL, which is caused by diverse *Leishmania* species with varying pathogeneses. Therefore, any extrapolation to other *Leishmania* species must be made with extreme caution, acknowledging the inherent specificity of the current model.

Future research should focus on optimizing AmB concentrations, release kinetics, and administration schedules, as well as thoroughly investigating local pharmacokinetic profiles and nanocarrier-induced immunomodulatory mechanisms. Validating these systems in other preclinical models that represent the diverse genetic and phenotypic presentations of different *Leishmania* species is essential to solidify their therapeutic potential and pave the way for clinical translation against CL.

## Data Availability

Data are available upon request and part of them are in the Supplementary Materials.

## Supplementary Materials

The following supporting information can be downloaded at: https://www.mdpi.com/article/10.3390/idr18010006/s1, Table S1: Data of the evolution of footpad lesions in mice infected with *L.* (*L.*) *amazonensis* treated after 30 days topically for 20 days with gel emulsion and PCL-AmB (~70 µg AmB).; Table S2: Data of the parasite load in footpad lesions and draining lymph nodes of BALB/c mice on day 30 post-infection determined by quantitative real-time PCR assays using DNA extracted from footpad and lymph node samples collected at the experimental endpoint (6 weeks post-infection) from mice infected with *L.* (*L.*) *amazonensis*; Table S3: Data of the evolution of footpad lesions in mice infected with *L.* (*L.*) *amazonensis* treated after 10 days topically for 10 with Oil_AmB and PCL-AmB (~70 µg AmB), alongside their respective controls; Table S4: Data of the parasite load in footpad lesions and draining lymph nodes of BALB/c mice on day 30 post-infection determined by quantitative real-time PCR assays using DNA extracted from footpad and lymph node samples collected at the experimental endpoint (40 days post-infection) from mice infected with *L.* (*L.*) *amazonensis*.
