# Supplementary material for: Beyond the Skin: Topical Amphotericin B Nanocarriers Targeting Cutaneous Leishmaniasis with Suppression of Lymphatic Parasite Burden
Source: Infect Dis Rep. 2026 Jan 6;18(1):6. doi: 10.3390/idr18010006 (PMC12821423; doi:10.3390/idr18010006)
Supplement: Supplementary file 1 [file idr-18-00006-s001.zip › idr-3955466-supplementary.pdf]

Table S1: Data of the evolution of footpad lesions in mice infected with L. (L.) amazonensis *treated after 30 days topically for 20 days with gel emulsion and PCL-AmB (~70 µg AmB).*

Days	Control_Oil	Gel_AmB	Oil_AmB
0	0,00 ± 0,00	0,00 ± 0,00	0,00 ± 0,00
14	0,03 ± 0,04	0,00 ± 0,00	0,00 ± 0,00
21	0,30 ± 0,12	0,44 ± 0,17	0,53 ± 0,07
30	0,45 ± 0,25	0,62 ± 0,12	0,65 ± 0,32
37	0,83 ± 0,30	0,84 ± 0,30	0,74 ± 0,30
44	1,50 ± 0,44	1,16 ± 0,27	1,06 ± 0,45
51	2,18 ± 0,69	2,12 ± 0,36	1,65 ± 0,52

The data are shown as means ± standard deviation of one experiment. Lesion size curves of treated groups were compared to those of the untreated control group (n = 5/group) by One-way ANOVA and no difference was observed.

Table S2: Data of the parasite load in footpad lesions and draining lymph nodes of BALB/c mice on day 30 post-infection determined by quantitative real-time PCR assays using DNA extracted from footpad and lymph node samples collected at the experimental endpoint (6 weeks post-infection) from mice infected with L. (L.) amazonensis.

	Foodpad	Lymph node
Control	20614,07 ± 13937,48	627,63 ± 30,65
Gel_AmB*	6580,23 ± 1962,80	772,63 ± 144,86
PCL_AmB*	1848,28 ± 555,61	347,25 ± 290,57

The data are shown as means ± standard deviation of one experiment. Lesion size curves for treated groups were compared with those of the untreated control group (n > 3/group). Statistical differences compared to the control were analyzed using the Student's t-test, where (*) corresponds to $p < 0.05$.

Table S3: Data of the evolution of footpad lesions in mice infected with L. (L.) amazonensis *treated after 10 days topically for 10 with Oil_AmB and PCL-AmB (~70 µg AmB), alongside their respective controls.*

Days	Control_Oil	Control_PCL	Oil_AmB	PCL_AmB
0	0,00 ± 0,00	0,00 ± 0,00	0,00 ± 0,00	0,00 ± 0,00
10	0,49 ± 0,26	0,56 ± 0,16	0,41 ± 0,05	0,29 ± 0,10
15	0,50 ± 0,00	0,60 ± 0,17	0,39 ± 0,11	0,36 ± 0,12
20	0,58 ± 0,22	0,58 ± 0,22	0,38 ± 0,29	0,37 ± 0,04*
30	1,15 ± 0,49	1,18 ± 0,74	0,41 ± 0,14**	0,36 ± 0,21*
35	1,10 ± 0,14	1,10 ± 0,14	0,15 ± 0,13***	0,35 ± 0,07***
40	1,40 ± 0,20	1,37 ± 0,15	0,38 ± 0,18**	0,65 ± 0,16*

The data are shown as means ± standard deviations from one experiment (n > 3/group). Lesion sizes were compared with the corresponding control groups using Student's t-test. Statistical significance: $p < 0.05$ (*), $p < 0.01$ (**), and $p < 0.001$(***).

Table S4: Data of the parasite load in footpad lesions and draining lymph nodes of BALB/c mice on day 30 post-infection determined by quantitative real-time PCR assays using DNA extracted from footpad and lymph node samples collected at the experimental endpoint (40 days post-infection) from mice infected with L. (L.) amazonensis.

	Foodpad			Lymph node		
Control_Oil	596,35	±	75,59	34,60	±	4,67
Oil_AmB	220,95	±	121,41*	10,63	±	3,03**
Control_PCL	541,90	±	1,41	23,40	±	11,17
PCL_AmB	798,93	±	1080,33	20,10	±	9,01

The data are shown as the mean ± standard deviation from two independent experiments (n > 3/group). Statistical significance compared to the control groups was analyzed using Student's t-test, $p < 0.05$ (*) and $p < 0.01$ (**).
